# Validity of the DISABKIDS^®^ - Cystic Fibrosis Module for Brazilian children and adolescents^1^

**DOI:** 10.1590/0104-1169.3450.2485

**Published:** 2014

**Authors:** Danielle Maria de Souza Serio dos Santos, Keila Cristiane Deon, Monika Bullinger, Claudia Benedita dos Santos

**Affiliations:** 2 PhD, Adjunct Professor, Faculdade de Farmácia, Universidade Federal do Rio de Janeiro, Campus Macaé, Macaé, RJ, Brazil; 3 PhD, Adjunct Professor, Escola Superior de Educação Física, Universidade Federal do Rio Grande do Sul, Porto Alegre, RS, Brazil; 4 PhD, Professor, Institut für Medizinische Psychologie, Universitätsklinikum Hamburg-Eppendorf, Hamburg, Germany; 5 PhD, Associate Professor, Escola de Enfermagem de Ribeirão Preto, Universidade de São Paulo, WHO Collaborating Centre for Nursing Research Development, Ribeirão Preto, SP, Brazil

**Keywords:** Quality of Life, Cystic Fibrosis, Validation Studies, Child, Adolescent

## Abstract

**OBJECTIVES:**

to validate the health-related quality of life measuring instrument DISABKIDS^®^ - Cystic Fibrosis Module (self version) for Brazilian children and adolescents.

**METHOD:**

methodological study in which a sample of 113 participants (54 girls and 59 boys; mean age 11.91 years and SD=2.79) was considered, from four Brazilian states, São Paulo, Paraná, Minas Gerais and the Federal District, 51 of whom participated in the pilot study and 62 in the field study. The answers to the questionnaire were analyzed, considering the frequency distributions with regard to the floor and ceiling effects, Cronbach's Alpha statistics, Pearson's Linear Correlation Coefficient, Mulitrait-Multimethod analysis and Confirmatory Factor Analysis according to Structural Equations Modeling.

**RESULTS:**

the instrument showed a high internal consistency coefficient (verified using Cronbach's Alpha) and construct validity, according to the Multitrait-Multimethod analysis. The DISABKIDS^®^ - Cystic Fibrosis Module, self version, maintained the same factorial structure as in the originally proposed model.

**CONCLUSION:**

the instrument validation has been finished and indicates that the self version is validated for use in Brazil and can be included into the monitoring routine of this population.

## Introduction

Cystic Fibrosis (CF) is a chronic condition and, until the start of the 20^th^ century, 80% of its patients did not get past the first year of life. As a result of advances in medical research, the genetic origin of CF was identified, with an autosomal recessive pattern, which causes a defect in chloride channels, present in the sudoriferous glands, respiratory, digestive and reproductive tract^(^ 1 ^)^. These findings, together with treatment advances, were associated with the increased life expectancy of its patients over the decades, allowing them to reach adult age and exposing the need to reconsider how health professionals should monitor this population, which is sometimes stigmatized as a result of singular characteristics like clubbed fingers and a barrel-shaped chest^(^ 2 ^)^.

Thus, health researchers and professionals become concerned with other aspects of these patients' life and health, moving beyond diagnostic issues, signs or symptoms. In this context, the Health-Related Quality of Life concept (HRQoL) is included in CF research. HRQoL is defined as a multidimensional concepts that considered physical, emotional, mental and social aspects related to health^(^ 3 ^)^. Although recent, the development and validation of specific HRQoL instruments for CF^(3-5) ^allowed psychosocial responses to this population's health problems to be truly considered as health measures in North American clinical research based on a publication by the regulatory agency Food and Drug Administration^(^ 6 ^)^. Some studies already indicate lower HRQoL scores among hospitalized children and adolescents with CF in the emotional and social and body image dimensions^(^ 7 ^)^, and that children who do not understand their condition and treatment can present a lower HRQoL score than others in the same condition^(^ 8 ^)^.

To use these instruments, however, they need to be available, i.e. adapted and validated for a certain culture or country. The construction of different instruments for the same area is advised against, as its process can be long and expensive and hamper the comparison between data from different populations. This limitation can be overcome by the use of existing instruments, based on the careful analysis of its cultural adaptation and validation process^(^ 9 ^)^.

The validation proposal of the DISABKIDS^®^ - Cystic Fibrosis Module (DISABKIDS^®^-CFM), the sole HRQoL instrument for CF exclusively for children and adolescents, is part of a partnership between the Ribeirão Preto College of Nursing (EERP-USP) and the University of Hamburg (UKE), Hamburg, Germany, where the coordination of the European DISABKIDS^®^(3) group is hosted, which works on the development of HRQoL measuring instruments for children and adolescents with chronic conditions. Its main characteristics are: rapid completion, demanding an average 15 minutes, and easily calculated and interpreted scores. The group has two generic modules, called the DISABKIDS^®^-Chronic Generic Module (DCGM) long-form (DCGM^®^-37) and short-form (DCGM^®^-12), besides specific modules for arthritis, asthma, atopic dermatitis, diabetes, epilepsy, cerebral palsy and the CF module. In Brazil, the DCGM^®^-37 and the specific atopic dermatitis module are going through the validation process^(10-12) ^and three new modules are being developed for children and adolescents with hearing impairment^(^ 13 ^)^, kidney disease^(14) ^and acquired immunodeficiency syndrome-AIDS^(^ 15 ^)^.

In view of the importance of working with well-defined and validated constructs for future assessments of the care process of children and adolescents with CF in Brazil, this study intends to present the validation data of the DISABKIDS^®^-CFM, self version, considering its psychometric properties, reliability, floor and ceiling effect, and to verify its factorial structure in order to check whether the HRQoL construct, in the elaboration of the instrument items, remains valid for Brazilian children and adolescents.

## Method

### Study design

Quantitative methodological study with cross-sectional design. Methodological research develops instruments and involves complex methods^(^ 16 ^)^. For instrument validation studies, the most important aspect is the verification of constructs or latent traits represented by observable behaviors, taking into account reliability and validity aspects of the instrument^(^ 17 ^)^.

### Place of study and period

The data was collected in two different periods, one for the pilot phase and another for the field study, totaling 113 children and adolescents and their respective parents or caregivers. In 2009^(^ 18 ^)^, the pilot phase took place, which involved 51 children and adolescents and their parents and caregivers. Next, between June 2011 and January 2013, the field study was carried out, involving another 62 participants, completing 113. The data was collected at outpatient clinics of referral centers for the treatment of CF patients from four Brazilian states, two in Curitiba - PR, one in Ribeirão Preto - SP, two in Brasília - DF and one in Belo Horizonte - MG. All outpatient clinics participants in the pilot and field phases.

The 51 children/adolescents and their respective parents or caregivers who participated in the pilot phase were considered in the final analysis, as there were no problems in the pilot study and its results were not used to calculate the sample size^(^ 18 ^)^.

### Ethical aspects

The study received approval from an Ethics Committee for Research Involving Human Beings (HCRP 6424/2008 and SES/DF 164/2011). All parents or caregivers who permitted the participation of their children and adolescents signed two copies of the Informed Consent Form, one of which was filed by the responsible researcher and the other by the parent/caregiver. It is emphasized that, even with the parents or caregivers' consent, only those children and adolescents who agreed to participate were included.

### Population and sample

The population consisted of Brazilian children and adolescents with CF, between eight and 17 full years of age. In order to participate, the children and adolescents could not be hospitalized and should possess cognitive skills compatible with their age. As regards to cognitive condition compatible with the age, no measuring instrument was used for this purpose. Instead, it was verified through reports from physicians, parents or caregivers.

For the sake of the study, at least 100 children and adolescents were considered, as this sample size permits the application of Confirmatory Factor Analysis (CFA) to the instrument^(^ 19 ^)^. A convenience sample was used, as the participants were contacted in order of arrival for their consultations at the clinics.

All of the children and adolescents answered the instruments separately and alone. Two children refused to participate in the study, one out of shyness and the other because she mentioned feeling unwell. Two mothers did not allow their children to participate, considering that they would be unable to answer the questionnaire.

### Instrument used

The DISABKIDS^®^-CFM has a self version for children and adolescents between eight and 17 full years of age, and a proxy version with the same items for parents or caregivers. Only the formulation of the items differs between the two instruments, allowing the parents or caregivers to answer the items thinking of their child or adolescent (E.g.: *Does your child get exhausted when she practices sports?*). This self-applied instrument consists of ten items that are easy to calculate. The dimensions assessed are called impact and treatment. The first includes four items and describes the feeling of fatigue and exhaustion. The second consists of six items and refers to the emotional impact of undergoing the treatment. The response alternatives are based on a five-point Likert scale, graded as follows: never, hardly ever, sometimes, often and always. For each dimension, a mean standardized score is obtained. This score ranges from 0% to 100%, in which 0% is associated with the most negative impact of the condition on the HRQoL and 100% with the least negative impact.

To use this instrument and all other modules of the DISABKIDS^®^ in Brazil, the European group authorizes and monitors the entire cultural adaptation and validation process of its instruments^(^ 10 ^-^ 15 ^,^ 18 ^)^.

### Data analysis

The scores were calculated according to the syntax of the DISABKIDS^®^-CFM, according to which there is no total score, but the scores of the impact and treatment dimensions are calculated separately. To be considered valid, the impact dimension, which includes four items, should be fully answered, while at least five out of six items should be answered in the treatment dimension, that is, there should be at least 83% of valid answers. One impact dimension was lost for one child, representing 0.9% of the sample.

The participants' distribution was described according to the answers, aiming to obtain the median, minimum, maximum and mean values and standard deviations, as well as to verify the existence of floor and ceiling effects. The latter two were considered present if more than 15% of the respondents chose the lowest or highest possible instrument score, respectively^(^ 20 ^)^.

The reliability of the instrument was measured using Cronbach's Alpha coefficient, which measures the internal consistency, and the test-retest, which measures its stability. To measure the internal consistency, Alpha coefficients between 0.70 and 0.95 were considered acceptable^(^ 20 ^)^. The retest was developed during three months, involving children and adolescents selected during the first application of the instrument through a draft (YES - will participate in the retest; NO - will not participate in the retest). To participate in the second application of the instrument, the following inclusion criteria were also considered: not having been hospitalized during this period and/or not having participated in any non-programmed consultation. The statistical test applied was the Intraclass Correlation Coefficient (ICC). Coefficients superior to 0.60 are considered acceptable^(^ 21 ^)^. The significance level used was 5% (α=0.05).

The construct validity of the instrument was measured according to its convergent and discriminant validity. The multitrait-multimethod (MTMM) analysis was used, which examines the correlations between items and dimensions. An appropriate software for this purpose is the Multitrait Analysis Program (MAP), which provides information about the allocation of the items in the scale and the scale fit for each of the items. The convergent validity is complied with if the correlation between an item and the dimension it belongs to is higher than 0.30 and, in final studies, than 0.40^(^ 9 ^)^. The discriminant validity, using the MAP, checks the percentage of times the correlation between an item and a dimension it belongs to is higher or statistically higher than its correlation with the dimension it does not belong to (fitness). Fitness coefficients close to 100% indicate the discriminant validity of the instrument.

The factorial structure of the DISABKIDS^®^-CFM was verified using CFA according to a Structural Equations Model (SEM). The model fitness was analyzed considering the Root Mean Square Error of Approximation (RMSEA) and the Comparative Fit Index (CFI). For the RMSEA, the approximation is good if its coefficient tends to zero, while values below 0.08 are acceptable, between 0.08 and 0.10 indicate median fitness and values superior to 0.10 weak fitness. The Comparative Fit Index was considered satisfactory with indices superior to 0.90^(^ 22 ^)^.

The results were described and analyzed using the software Statistical Package for Social Sciences (SPSS), version 19.0. The module Analysis of Moment Structure (AMOS), version 19.0 (License 10101111255, 09/14/2011), was used for the CFA of the DISABKIDS^®^- CFM.

## Results

The final sample consisted of 113 children and adolescents (54 girls and 59 boys), with a mean age of 11.91 years (SD=2.79), 51 of whom were part of the pilot study and 62 of the field study. As regards the parents and caregivers, the mean age was 41.05 years (SD=8.12) and 80.5% of the respondents were the mothers of the children and adolescents. Twenty children were interviewed from the referral center in Ribeirão Preto, 18 from Belo Horizonte, 35 from the centers in Brasília and 40 from the centers in Curitiba.

Table 1 presents the descriptive results from the validation for Brazil in comparison with the data found in the original validation.

**Table 1:** - Standardized means, medians and standard deviations for the DISABKIDS^®^-CFM for children and adolescents who participated in the Brazilian study in comparison with the values found in the European study. Brazil, 2013.

Dimension	N (Brazil/Europe)	Mean (Brazil/Europe)	Standard Deviation (Brazil/Europe)	Median (Brazil)
Impact (0-100)	112/26	72,71/66,83	20,34/20,14	75,00
Treatment (0-100)	113/28	67,70/68,37	23,23/24,14	66,67

The presence of a ceiling effect (16.8%) was verified in the impact dimension of the self version.

As regards the internal consistency, the impact dimension showed an Alpha coefficient of 0.71, against 0.76 for the treatment dimension.

The retest of the instrument involved 17 children and adolescents. The ICC coefficients corresponded to 0.505 (p=0.011) for the impact dimension and 0.480 (p=0.020) for the treatment dimension.

Concerning the convergent validity, Table 2 displays the Pearson's correlation coefficients between the items and each of the dimensions according to the MTMM analysis.

**Table 2:** - Pearson's correlation coefficients between the items and each of the dimensions of the DISABKIDS^®^ - CFM self version, according to MTMM analysis, Brazil, 2013.

Item	Impact	Treatment
01	0,48	0,34
02	0,54	0,14
03	0,51	0,22
04	0,48	0,28
05	0,05	0,26
06	0,29	0,37
07	0,28	0,60
08	0,31	0,57
09	0,23	0,63
10	0,21	0,61

For the discriminant validity, the self version showed 100% fitness, that is, all items displayed higher and significantly higher correlations with their respective dimensions than their correlation with the other dimension.

**Figure 1:**
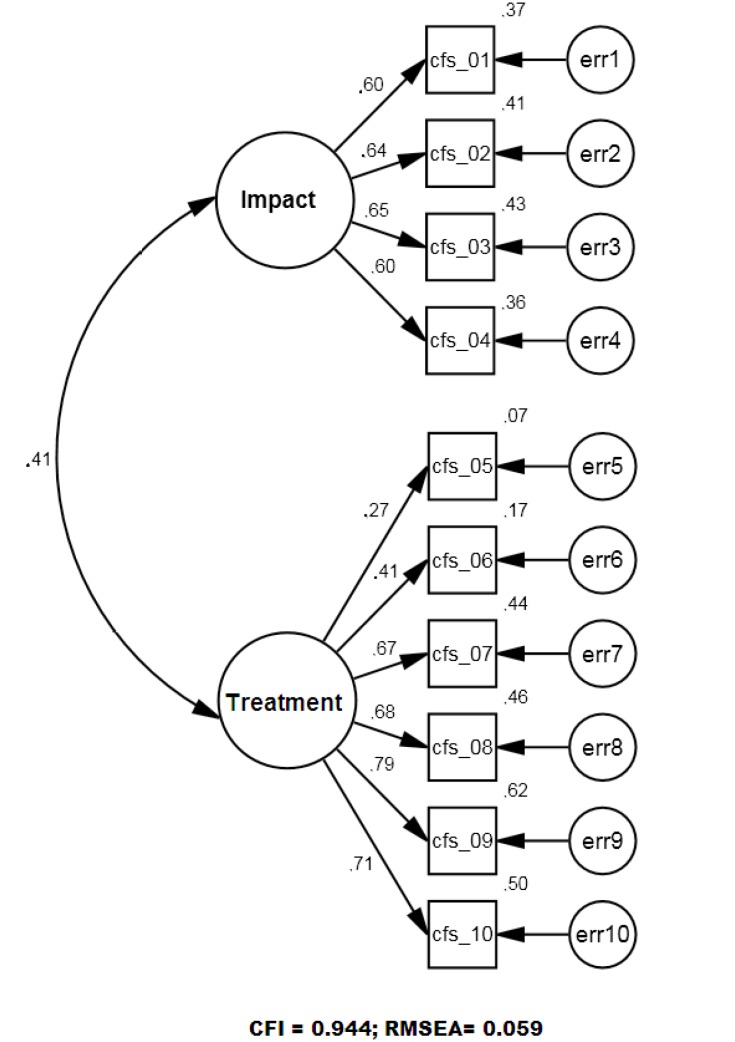
- Confirmatory Factor Analysis of the DISABKIDS^®^ - CFM self version. Brazil, 2013.

Figure 1 shows the CFA of the self version of the DISABKIDS^®^-CFM.

## Discussion

The use of the HRQoL measure adds the patients' perspective in their treatment and other subjective health aspects involved in their lives. This is clearly important for CF, as this knowledge is still developing with the aging of the population^(^ 23 ^-^ 24 ^)^.

The mean and median values found in the impact and treatment dimensions of the self version are superior to the scale averages. As these data are not standardized in Brazil, these values are but descriptive.

The Cronbach's Alpha coefficients found were substantial and indicate the internal consistency of the instrument, in that all of its items, in the respective dimensions, are measuring the same latent trait^(^ 9 ^,^ 20 ^)^.

The presence of a ceiling effect in the impact dimension of the self version can be related to the considerations by other researchers who indicate that patients with CF tend to choose the highest possible score in a HRQoL instrument^(^ 25 ^)^ and can mention the ability to adapt to their health reality^(^ 25 ^)^. Once present, caution is due for the ceiling effect not to limit the instrument's responsiveness, as changes over time may not be attributed to the interventions but to the presence of this effect^(^ 9 ^)^.

In the test-retest analysis, the ICC coefficients remain below the ideal levels. These results may be attributed to the long period the retest was applied as, ideally, the instrument should be reapplied between one and two weeks^(^ 9 ^,^ 20 ^)^. The decision to assess these coefficients over this long period is justified by the data collection difficulty, due to the dynamics of care at the outpatient clinics. Most patients have a scheduled return appointment every three months (and only more severe cases return more frequently) and many of them come from outside the city where the care institution is located. As an important measure to interpret individual changes that occur over time, before using the DISABKIDS^®^-CFM in intervention studies, this information should be reassessed, using the ideal retest time.

For the convergent validity, it was observed that the correlation between each item and its respective dimension in most cases was superior to 0.40, except for item 5 (r=0.26). Although inferior to 0.40, item 6 (r=0.37) remains within the range of satisfactory coefficients^(^ 9 ^)^. Thus, with satisfactory convergent and divergent validity coefficients (100% adjustment), the instrument shows construct validity.

The CFA, applied to check the adjustment of the final model achieved through the cultural adaptation of the DISABKIDS^®^-CFM, indicated that the adapted version for children and adolescents (*self*) maintained the factor structure of the original instrument, with RMSEA and CFI coefficients that indicate that the meaning of the items in the study context was maintained, that is, that the adapted version for children and adolescents measures the original construct of the instrument.

## Conclusion

The DISABKIDS^®^-CFM to measure the HRQoL of Brazilian children and adolescents with CF has been validated and indicates that the self version is valid for use in Brazil.

The adaptation and validation of a specific HRQoL measuring instrument for CF, exclusively developed for children and adolescents, guarantees to the researchers that the application of the instrument among the participants assessed the same construct.

In view of its easy completion, the ten-item self version of the DISABKIDS^®^-CFM, now validated for Brazil, can be included into the routine monitoring of this population, without compromising care delivery to these patients and the time available for treatment.

In addition, as part of a development project of HRQoL instruments for children and adolescents, based on a standardized theoretical-methodological framework, when available in Brazil, these instruments can be used in combination, permitting studies to assess and compare this population's HRQoL with others suffering from some chronic conditions.
